# Real-Time Mass Spectrometry Monitoring of Oak Wood Toasting: Elucidating Aroma Development Relevant to Oak-aged Wine Quality

**DOI:** 10.1038/srep17334

**Published:** 2015-11-27

**Authors:** Ross R. Farrell, Marco Wellinger, Alexia N. Gloess, David S. Nichols, Michael C. Breadmore, Robert A. Shellie, Chahan Yeretzian

**Affiliations:** 1 Australian Centre for Research on Separation Science (ACROSS), University of Tasmania, Hobart, Tasmania 7001, Australia; 2 Zurich University of Applied Sciences, Institute of Chemistry and Biological Chemistry, 8820 Wädenswil, Switzerland; 3 Central Science Laboratory, University of Tasmania, Private Bag 74, Hobart, Tasmania 7001, Australia

## Abstract

We introduce a real-time method to monitor the evolution of oak aromas during the oak toasting process. French and American oak wood boards were toasted in an oven at three different temperatures, while the process-gas was continuously transferred to the inlet of a proton-transfer-reaction time-of-flight mass spectrometer for online monitoring. Oak wood aroma compounds important for their sensory contribution to oak-aged wine were tentatively identified based on soft ionization and molecular mass. The time-intensity profiles revealed toasting process dynamics illustrating in real-time how different compounds evolve from the oak wood during toasting. Sufficient sensitivity was achieved to observe spikes in volatile concentrations related to cracking phenomena on the oak wood surface. The polysaccharide-derived compounds exhibited similar profiles; whilst for lignin-derived compounds eugenol formation differed from that of vanillin and guaiacol at lower toasting temperatures. Significant generation of oak lactone from precursors was evident at 225 ^o^C. Statistical processing of the real-time aroma data showed similarities and differences between individual oak boards and oak wood sourced from the different origins. This study enriches our understanding of the oak toasting process and demonstrates a new analytical approach for research on wood volatiles.

Oak wood has long been used in wine aging to enhance wine aroma, taste, colour and stability^1^,^2^. Seasoning and toasting of oak products intended for wine-aging are important to produce oak wood of suitable sensory quality^1^. Freshly sawn oak wood is seasoned in the open air to leach astringent ellagitannins, hydrolyse bitter glycosylated coumarins and increase aroma compounds due to degradation of wood macromolecules by microflora^3^,^4^,^5^,^6^. Toasting results in a dramatic alteration of wood chemistry through hydrothermolysis and pyrolysis reactions^1^. Volatile organic compounds produced by thermal degradation of polysaccharides, lignin, and lipids greatly affect the sensory quality of oak-aged wine^7^,^8^,^9^,^10^.

It is important to understand the chemistry of oak toasting, but previous studies are difficult to compare due to the diversity of extraction methods, the size of the wood samples (chips, boards, barrels), and the use of qualitative descriptions of the toasting process rather than explicit statements of temperature and time^11^. Furthermore, several publications have reported contradictory findings regarding the impact of toasting temperature on the development of oak aroma^11^.

It has been difficult to investigate the dynamic process of oak toasting using traditional offline approaches, so limited studies have investigated temporal changes in oak wood chemistry with toasting. Currently, chemical analysis of oak wood volatiles is performed offline using gas chromatography following lengthy sample preparation^11^,^12^,^13^,^14^,^15^,^16^,^17^. Consequently, discrete toasting treatments (one temperature, one toasting period) are currently required for each time period of interest, limiting existing studies to two different time periods for a given toasting temperature^8^,^11^,^13^.

Different mass spectrometry approaches can be envisaged for real-time monitoring of the oak toasting process. Briefly, these may include proton-transfer-reaction mass spectrometry^14^, and laser based methods employing photoionization, including resonance enhanced multiphoton ionization mass spectrometry^15^, and single photon ionization mass spectrometry^16^.

Given the availability and prior success of proton-transfer-reaction mass spectrometry approaches for online monitoring of the coffee roasting process^17^,^18^,^19^, here we take a similar analytical approach. By using online mass spectrometry we illustrate the analysis of oak toasting in real-time. This is achieved by continuous monitoring of volatile compounds during the toasting process. Continuous, real-time data clarify oak aroma development during the toasting process, and generate a new quality of information that is impossible to obtain using the traditional offline approaches. We combine the mass spectrometry data with chemometric protocols to examine differences between individual oak boards and differences between French and American oak.

## Results and Discussion

### Wood Properties

Oak toasting is a process whereby the wood is brought to a temperature in the range of 150–240 °C^11^ for a certain duration. It can be generally divided into two phases: a drying phase, during which water is removed from the hydrophilic wood constituents (hemicellulose and cellulose) and a toasting phase were complex thermal degradation reactions occur transforming non-volatile precursors into aroma active volatile compounds. The moisture content (Supplementary Table 1) of the oak samples varied minimally, both having average moisture content of 10% (w/w), with standard deviations <0.94%. Wood density can also affect wood heating rate^20^ and energy absorption^21^. However, we found no significant difference (p > 0.05, two-tailed t-test) between the oak sources for the small sample size (n = 4) used here (Supplementary Table 1).

### Compound Identification

The measured and theoretical monoisotopic masses for the target compounds, their chemical formulae, tentative identification, corresponding mass errors, and total peak areas are reported in Table 1.

### Repeatability Experiments

**Figure 1:**
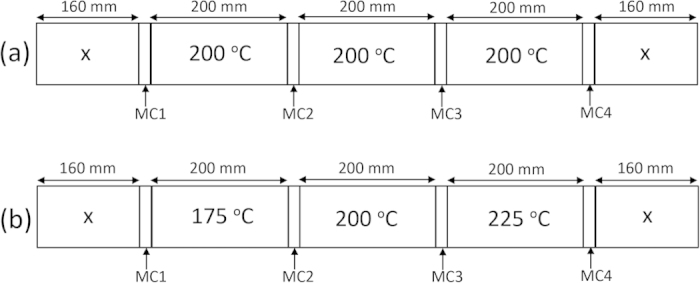
Cutting pattern applied to each board and toasting temperature applied to each sample. (**a**) Protocol for repeatability experiments. (**b**) Protocol for toasting temperature experiments. X: sections discarded; MC: sections used for analysing moisture content and density.

**Figure 2:**
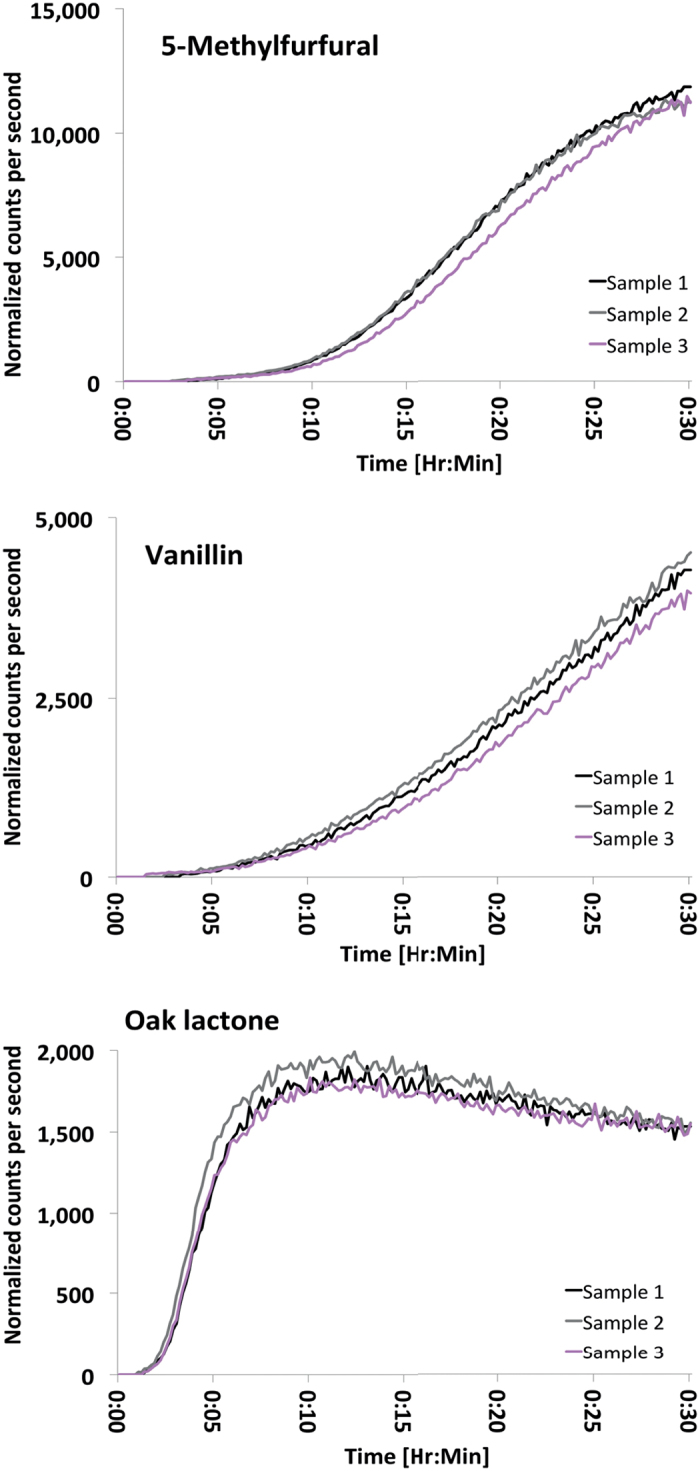
Time-intensity profiles for three compounds evolving from different aroma precursors in American oak wood, showing reproducibility of measurements based on three samples from the same board toasted at 200 ^o^C. Normalised counts per second (ncps) are plotted against toasting time.

**Figure 3:**
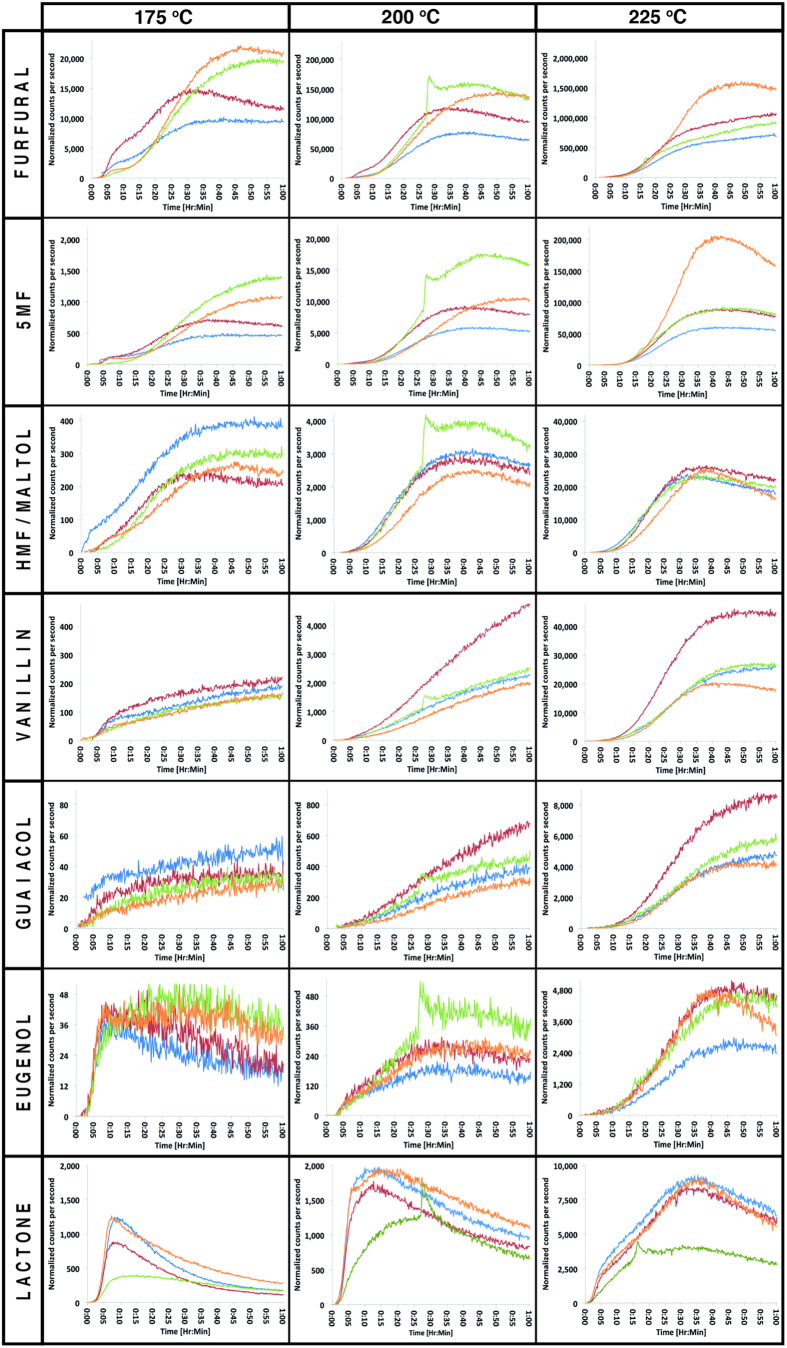
American oak time-intensity profiles for the target compounds at three different temperatures for individual board samples (Board 1: blue, Board 2: red, Board 3: green, Board 4: orange). Normalised counts per second (ncps) are plotted against toasting time. Note that y-axis scaling increases by one order of magnitude for each increase in toasting temperatures.

The oak samples taken from the same board and toasted at the same temperature (protocol (a) in Fig. 1), produced similar time-intensity profiles for the ion traces we studied. Figure 2 shows the time-intensity profiles for the American oak samples taken from the same board. Compared with the American oak profiles for samples taken from different boards (Fig. 3), it is clear that between-board variation exceeds within-board variation. The peak area relative standard deviations for within-board samples compared to between-board samples (calculated at 30 minutes) were 6% versus 41%, 9% versus 37% and 4% versus 23% for 5-methylfurfual, vanillin, and oak lactone, respectively. Accordingly, the dimensions of the sample (200 × 50 × 10 mm) appear large enough to provide three samples representative of each board. Repeatability and precision of the approach is evident in that analysis of samples from the same board result in consistent profiles with a relative standard deviation <10%. Consequently, observed differences in the aroma profiles can be assigned to differences in oak properties and toasting temperatures, rather than to variability of the analytical method.

### Oak aroma time-intensity profiles

**Figure 4:**
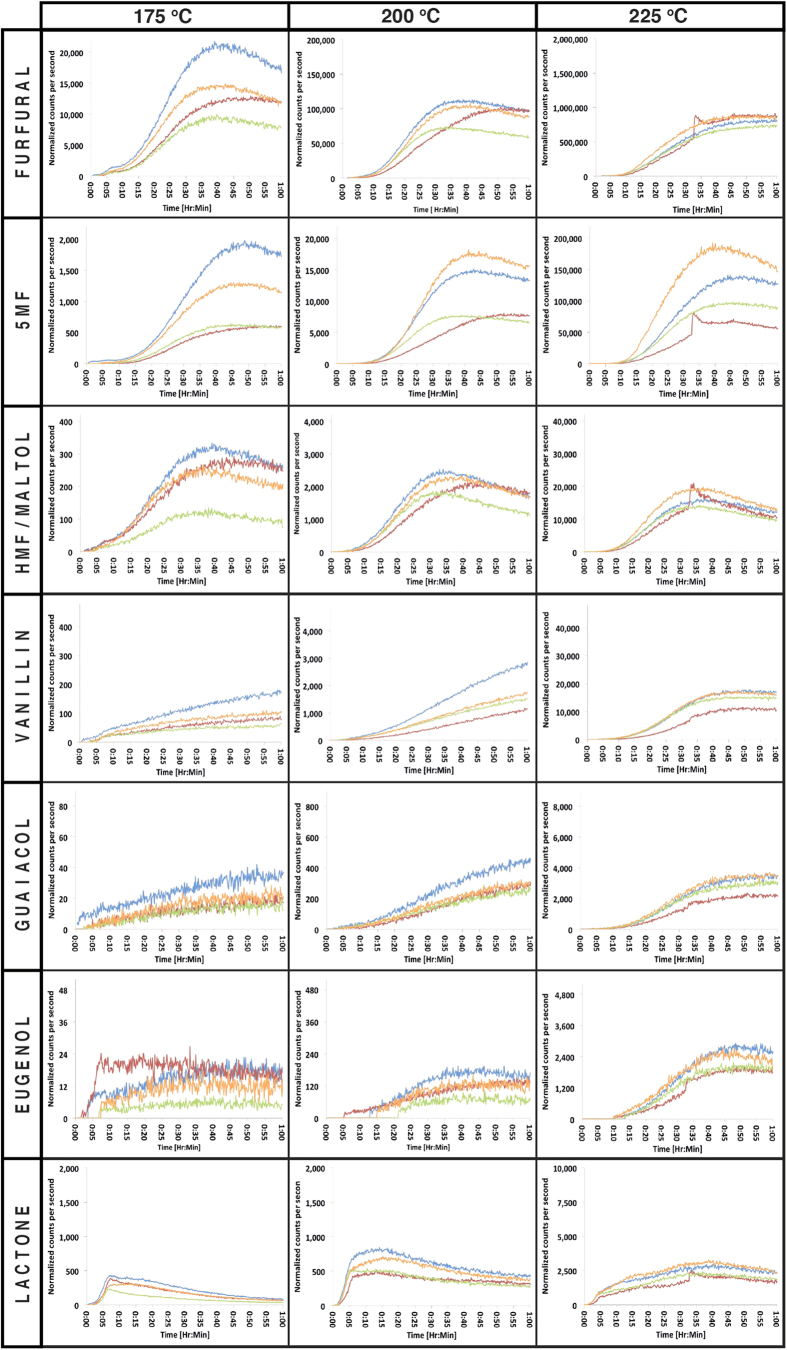
French oak time-intensity profiles for the target compounds at three different temperatures for individual board samples (Board 1: blue, Board 2: red, Board 3: green, Board 4: orange). Normalised counts per second (ncps) are plotted against toasting time. Note that y-axis scaling increases by one order of magnitude for each increase in toasting temperatures.

The time-intensity profiles shown in Figs 3 and 4 for both American and French oak samples respectively show the dynamic evolution of the selected oak volatiles during toasting. It is important to emphasize that the measured time-intensity profiles reflect the combined effect of three processes; (a) generation of volatiles from precursors, (b) diffusion of volatiles through the physical wood structure and (c) volatilisation from the wood surface. The time-intensity profiles highlight differences between boards and the oak sources. It is notable that the sudden increase in compound levels observed at approximately 30 min for two of the samples (American oak board three at 200 ^o^C and French oak board two at 225 ^o^C) relates to a physical and audible crack in the wood surface. This crack facilitates rapid escape of hot gas from within the wood structure causing a sudden spike in the concentration of the monitored volatiles. A similar phenomenon is observed in the coffee roasting process where spikes in compound concentrations correspond with the first and second “crack”, as cells rupture under high internal pressure^17^. American oak board three, toasted at 225 ^o^C also displayed evidence of cracking at 15 min.

### Polysaccharide-derived compounds

The time-intensity profiles for furfural, 5-methylfurfural (5MF) and 5-hydroxymethylfurfural (HMF)/maltol reported in Figs 3 and 4 were similar for each toasting temperature. Concentrations of oak volatiles remained low until 10–15 min of toasting (shorter for higher temperatures) and then increased to a maximum, followed by a decrease. The rate of decrease was greater at higher temperatures. Whilst the shape of the time-intensity profiles remained similar for all temperatures, maximum concentrations increased by approximately one order of magnitude with each 25 °C increase in toasting temperature.

The furfural peak was clearly the most intense signal observed for the selected volatiles, indicating that furfural is generated and volatilized in high quantities during the toasting process. Furanic compounds are produced by depolymerization of hemicellulose catalyzed by acetic acid (another compound observed at high intensities) and is formed during heating of wood^11^. Hemicellulose is the most thermo-sensitive wood polymer due to reactive acetyl side groups that are highly susceptible to acid hydrolysis, degrading in the temperature range of 130–194 ^o^C^21^. When oak wood is heated, pentoses (the main constituents of hemicelluloses) produce furfural, thus explaining the high signal-intensities observed for furfural as toasting progresses. In contrast, 5-methylfurfural and 5-hydroxymethylfurfural/maltol are generated from hexose sugars, a minor constituent of oak wood hemicellulose and mainly found in the more crystalline cellulose^22^,^23^. The high degree of crystallinity^24^,^25^ provides resistance to acid hydrolysis at the relatively low temperatures (150–240 °C^11^) used during oak toasting, hence these compounds would be expected at much lower intensities in toasted oak relative to furfural concentrations. This can be confirmed by the lower intensities observed for these compounds in this study, as shown in Fig. 3 and Fig. 4. Furthermore, the higher boiling point of 5-hydroxymethylfurfural/maltol (292 ^o^C and 285 ^o^C respectively) versus that of 5-methylfurfural (187 ^o^C) may influence their relative intensities.

### Lignin-derived compounds

Time-intensity profiles for vanillin, guaiacol and eugenol (Figs 3 and 4) differed according to temperature. Vanillin and guaiacol had similar profiles, whilst the eugenol profile was different, except at 225 ^o^C where the profile was similar to that of vanillin and guaiacol. At 225 ^o^C, the vanillin and guaiacol profiles were similar to the profile of furanic compounds. In accordance with previous findings the time-intensity profile for eugenol is different from that of vanillin and guaiacol^11^: Eugenol levels remained low at 175 ^o^C (close to background noise levels) and increased to a maximum at 5–10 min. For French oak, levels remained stable, in contrast to the decreasing intensity trend observed for American oak samples. At 200 ^o^C, signal intensity increased until approximately 40 min. Thereafter, a gradual decrease was observed with time. A similar but more distinct profile was observed at 225 ^o^C.

Although lignin is generally considered to be the most thermally stable wood polymer (degrading at 280–500 ^o^C)^25^, some degradation of lignin has been shown to occur at relatively low temperatures (e.g. 165 ^o^C)^26^. This is consistent with our results, where the concentrations of lignin-derived compounds (vanillin, guaiacol and eugenol) clearly increase with temperature, by approximately one order of magnitude for each 25 °C increase in toasting temperature, as illustrated in Fig. 3 and Fig. 4. Considering the volatility of these compounds (boiling point of vanillin 283 ^o^C, guaiacol 205 ^o^C and eugenol 255 ^o^C) we observe the highest intensities for vanillin (at all temperatures) even though vanillin has the lowest volatility. This confirms a higher concentration of vanillin relative to the other lignin-derived compounds.

### Lipid-derived compound oak lactone

Temperature had a notable effect on the shape of the time-intensity profiles for oak lactone, particularly considering the differences between profiles at 175 ^o^C and 225 ^o^C (Figs 3 and 4). At 175 ^o^C, lactone intensity increased rapidly (0–6 min), and then decreased with time. At 200  ^o^C, the initial rapid increase was again evident, followed by a second (moderate) increase until approximately 15 min. Signal intensities then decreased steadily with time. At 225 ^o^C, the initial rapid increase was followed by a notable increase that continued and reached a maximum at approximately 35–40 min. Lactone signal intensity then decreased over time, more slowly than the rate of decrease observed at 175 ^o^C and 200 ^o^C. For American oak, board three had notably lower lactone intensity across all investigated temperatures. Furthermore, the profile deviated from the trend observed for the other American oak boards. Compared to the compounds derived from polysaccharides and lignin, there was a less pronounced increase in signal intensity with increased temperature.

Toasting treatments have been reported to have different effects on lactone levels (no effect, decreasing at high temperature, increasing then decreasing, or to decrease then increase)^11^. Recently, Wilkinison^13^ helped clarify the formation of oak lactone by investigating the thermodegradation of oak lactone glycoconjugate precursors. They examined precursor levels at two toasting temperatures (100 ^o^C and 200 ^o^C) conducted for two different time periods (5 and 30 min). Their results showed thermal degradation of the lactone precursor (implying generation of volatile free lactone), but only after toasting at 200 ^o^C for 30 min. In the current study we observe that oak lactone signal intensities exceed 175 ^o^C levels after 10 min of toasting at 200 ^o^C, reaching a maximum after 15 min. Lactone intensities at 200 ^o^C are approximately double that observed at 175 °C. This increase in lactone at 200 °C could relate to greater volatilization of free-lactone (boiling point 247 ^o^C), increased diffusion from deeper in the board sample and/or to the generation of lactone from the precursors (as indicated by Wilkinson^13^).

At 225 ^o^C the maximum signal intensity is approximately 5–10 times higher than that observed at 175 °C (for French and American oak, respectively). The remarkably different time-intensity profile observed at 225 ^o^C supports the view that high quantities of free lactone are generated from precursors at temperatures above 200 °C.

### Between-board variation

The time-intensity profiles described above (Figs 3 and 4) also illustrate differences in the volatilized compounds between individual oak boards. For example, comparing the boards with the highest and lowest lactone peak areas, the greatest difference was observed at 175 ^o^C, 224% for American oak, and 249% for French oak. 5-methylfurfural showed the highest between-board variability with relative standard deviations as high as 55%. The lowest variation was observed for 5-hydroxymethylfurfural/maltol with relative standard deviations as low as 7%. In general the ranking of a board changed according to the compound. For example, American oak board 1 (blue) was ranked high for oak lactone and 5-hydroxymethylfurfural/maltol, intermediate for vanillin and guaiacol and low for 5-methylfurfural, furfural and eugenol. These findings show that individual oak boards may react differently under similar toasting conditions, even when the analysis is limited to boards of the same origin, moisture content and density.

## Statistical Analysis

### Toasting Effects

According to their relative contents (area under the curve after one hour of toasting) the ANOVA for all 24 samples (Table 1) showed that the temperature effect was highly significant for all compounds. However, 5-hydroxymethylfurfural/maltol was the only compound that showed significant differences between all three toasting temperatures (Supplementary Table 2). The other compounds were differentiated by two groups, one related to the 225 ^o^C toasting treatment (high compound concentrations), and another grouping the 200 ^o^C and 175 ^o^C treatments (exhibiting lower compound concentrations).

### Oak Source Effects

With the exception of furfural and 5-methylfurfural all compounds were significantly higher in American oak (Tukey’s pairwise comparisons, Supplementary Table 3 – Supplementary Table 9). As reported in Table 1 there was a significant interaction effect between oak source and temperature for most compounds (excluding furfural and 5-methylfurfural), i.e. the toasting effect depended on the oak source for most of the compounds we studied. Caldeira *et al.*^22^ also reported similar results for a “wood origin” effect for the compounds targeted in the current work.

### Oak Lactone Content

The oak lactones are among the most important volatile compounds with a high sensory impact on oak-aged wines^27^. In agreement with the documented literature we observed significantly higher quantities of oak lactone in the American oak samples (Supplementary Table 9)^28^. Oak boards could be separated into three groups according to their oak lactone quantities (Tukey’s pairwise comparisons, Supplementary Table 10): American oak 225 ^o^C with high levels, French oak 175 ^o^C with low levels and the remaining categories displaying “medium” lactone levels.

### Principal Component Analysis

**Figure 5:**
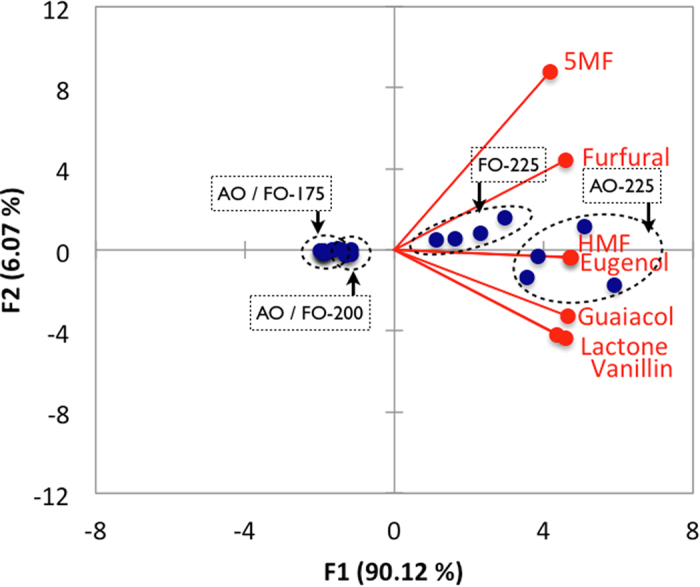
Principal component analysis score and loadings plot for first and second components (F1 and F2) for all oak categories and variables. Note, 5MF refers to 5-methylfurfural and HMF to 5-hydroxymethylfurfural/maltol. The dashed ellipses are used to visualise the species and temperature groupings and do not represent a confidence interval.

The principal component analysis for all oak categories (i.e. oak source and toasting temperatures) presented in Fig. 5 demonstrated little separation between the 175 ^o^C and 200 ^o^C toasting treatment. However, the 225 °C treatmentwas clearly differentiated from the others and separation of the oak source (i.e. French from American oak) was also evident at this temperature. The first principal component (F1), accounting for 90% of the total variance, relates to the increase in compound intensity with increasing temperature. F1 is strongly associated with 5-hydroxymethylfurfural/maltol and eugenol, and separates low temperature treatments (175 ^o^C and 200 ^o^C) from the 225 ^o^C toasting treatment. F1 also provides separation between the oak sources with American oak exhibiting higher signal intensities. The second principal component (F2) accounts for only 6% of the variation separating boards that are high in furanic compounds (particularly 5-methylfurfural and furfural) versus those high in vanillin and oak lactone. Caldeira *et al.*^22^ observed separation of oak species based on principal component analysis of the volatile composition for untoasted wood, but not when toasted samples were included in the analysis. They did not observe separation based on toasting level, only separating untoasted from toasted samples. Their study used traditional barrel toasting over open fire however with no disclosure of the toasting temperature applied. The “strong” toast they applied was 25 min compared to the 60 min applied herein. Furthermore, the oven toasting method used in the current work (as opposed to standard industry toasting of barrels over open fire) would likely provide better control of the heat applied to the board samples, reducing variation at a given temperature. Probably, a combination of a longer toasting period, potentially higher temperature and greater control of the toasting process in the current work helps separate the “high” versus “low” temperature toasts. The principal component analysis shows that the 175 ^o^C and 200 ^o^C toasting treatments produce toasted oak boards with similar aroma profiles in contrast to clear differences observed when the toasting temperature is increased to 225 ^o^C.

### Agglomerative Hierarchical Clustering Analysis

**Figure 6:**
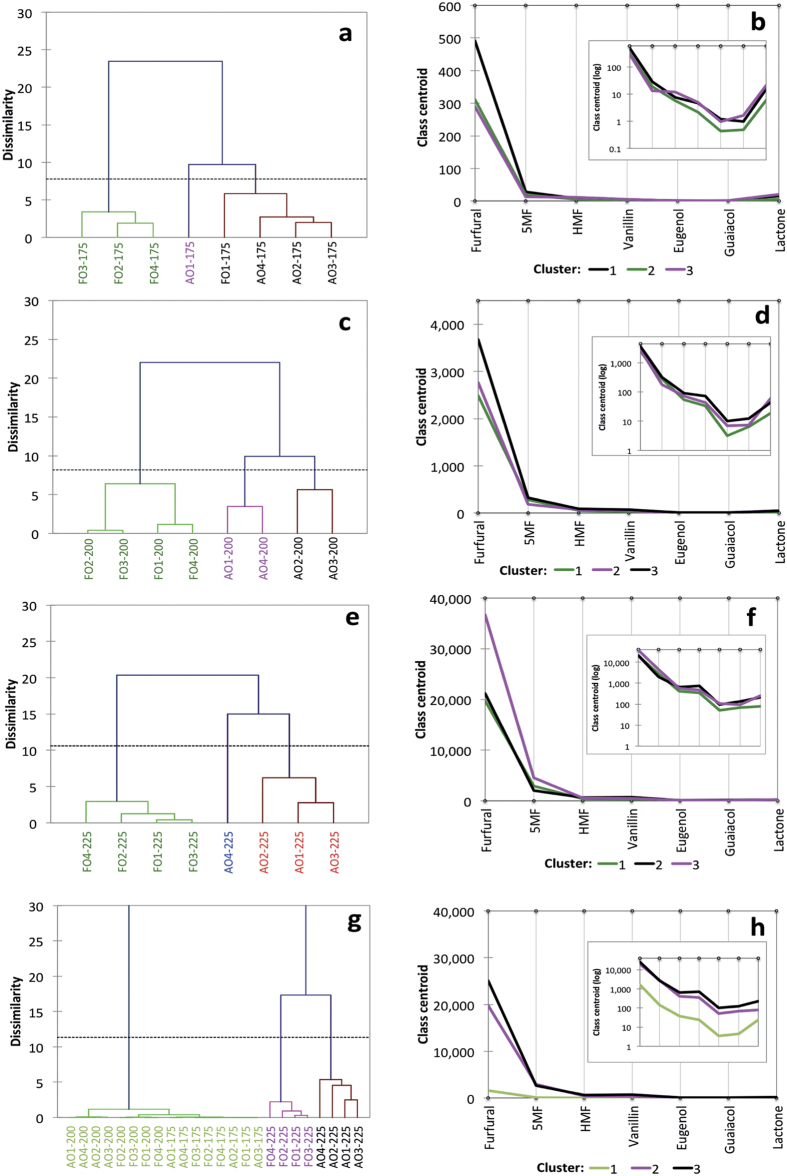
Agglomerative hierarchical clustering dendrogram (**a,c,e,g**) and compound profile plots (**b,d,f,h**) showing the aroma composition associated with each cluster (including a log-scale inset). All plots are based on integrated peak area after one hour of toasting.

Considering toasting temperatures individually (Fig. 6a,c,e), agglomerative hierarchical clustering grouped the eight oak boards into three clusters at each temperature. However, the composition of the three clusters varied depending on the temperature. At 175 ^o^C (Fig. 6a) French oak boards were clearly separated from American oak, with the exception of French oak Board 1 that was classified with the American oak. American oak Board 1 was placed in a unique, separate cluster. The profile plot (Fig. 6b) showed that this cluster was characterized by low levels of furfural, and high levels of guaiacol and lactone. American oak boards were differentiated from the French oak by higher concentrations of the selected volatiles. At 200 ^o^C French oak boards were separated from the American oak (Fig. 6c), again generally based on higher compound concentrations in American oak. The American oak boards were separated into two groups based on higher levels of oak lactone and lower levels of the other compounds (Fig. 6d). At 225 ^o^C, once again French oak boards were separated from American oak (Fig. 6e), whilst American oak Board 4 was differentiated from the other American oak boards primarily based on high furfural and 5-methylfurfural levels (Fig. 6f).

When agglomerative hierarchical clustering was applied to all boards (across all temperatures) three clusters were identified (Fig. 6g); one grouping all boards toasted at 175 ^o^C and 200 ^o^C, a second grouping the French oak boards toasted at 225 ^o^C and a third grouping all American oak boards toasted at 225 ^o^C. This was further demonstrated by the Tukey’s test pairwise comparisons (Supplementary Table 10) given the statistical differences between the American oak 225 ^o^C treatment and the others. Considering these findings, we show that oak boards can be readily classified according to their toasted aroma profiles using chemometric tools.

In summary, we have demonstrated a real-time methodology for online monitoring of the oak toasting process based on PTR-ToF-MS. The method requires no sample preparation and entails direct introduction of the oak toasting process gas into the inlet of the mass spectrometer. The results presented show the utility of PTR-ToF-MS for monitoring the changes in aroma active volatiles during the oak toasting process. Real-time monitoring revealed how aroma compounds derived from different precursors develop over time. This online method circumvents limitations associated with previous gas chromatography based approaches by (i) greatly improving the temporal resolution (one measurement was recorded every 2 s) allowing the time-intensity profile for key aroma compounds to be directly monitored in real-time, (ii) avoiding liquid extractions and lengthy sample preparation protocols, (iii) reducing sources of error associated with liquid extractions (iv) avoiding multiple time-discrete toasting treatments by elucidating the time dimension in a single dynamic experiment. In addition, proton-transfer-reaction mass spectrometers are field-deployable^29^, hence this approach is relevant to both laboratory-based and industry-based (*in situ*) research. Portable instrumentation offers a potential route to optimization of (site-specific) toasting systems to achieve targeted aroma profiles.

The combination of real-time monitoring, precise specification and control of toasting temperatures applied in this study helps elucidate the development of important oak aroma compounds during the toasting process. Our observations conducted in real-time also highlight the variable nature of oak wood chemistry. Individual oak boards react differently under similar toasting conditions, even when the analysis is limited to boards of the same origin, moisture content and density. Natural variation in oak chemistry is well documented in the prior research conducted offline, using gas chromatography approaches^10^,^11^,^30^,^31^,^32^. Combined with chemometric approaches the described PTR-ToF-MS methodology can be used to differentiate or group similar oak boards according to their toasted aroma profiles.

In this work, we provide new insights into the dynamics of the oak toasting process. We anticipate that real-time mass spectrometry approaches could readily be used in many different applications to further our understanding and control of oak wood flavor chemistry, improving the sensory quality of oak-aged wine, spirits and beer.

## Methods

### Oak samples

Seasoned, non-toasted oak boards classified only by source (American oak from Kentucky and French oak from forests of central France) were provided in “Fan-assembly” form by Canton cooperage (Lebanon, KY, USA). The French oak boards were sawn lengthwise to 50 mm widths to match the dimensions of the American oak boards (10 × 50 × 980 mm). Subsequently, 160 mm sections were cut from each end of the board and discarded (“X” in Fig. 1), to improve homogeneity by avoiding sampling from the board ends. Three 200 mm board samples were then cut taking 15  mm wide sections between each sample for determination of sample board moisture content determined by the oven dry method (MC 1-4), Fig. 1 33. The moisture content samples were also used to calculate density based on the oven dry weight and oven dry volume, with volume determined by the water immersion method^34^.

### Oak toasting experiments

Firstly, three samples from a single American and French oak board were toasted at the same temperature as shown in protocol (a) in Fig. 1. This protocol was used to test the repeatability of the experimental method and to examine potential variation between samples within a given board. Secondly, to investigate the temperature effect, one sample from each of the four boards (for both French and American oak) was toasted at each temperature (175 ^o^C, 200 ^o^C and 225 ^o^C) for 1 h as shown in protocol (b) in Fig. 1.

### Experimental setup

Toasting was conducted in a 53 L forced convection oven (model FD-53, Binder GmbH, Tuttlingen, Germany). The oven was preheated to the desired toasting temperature before placing the oak board sample on the middle rack.

**Figure 7:**
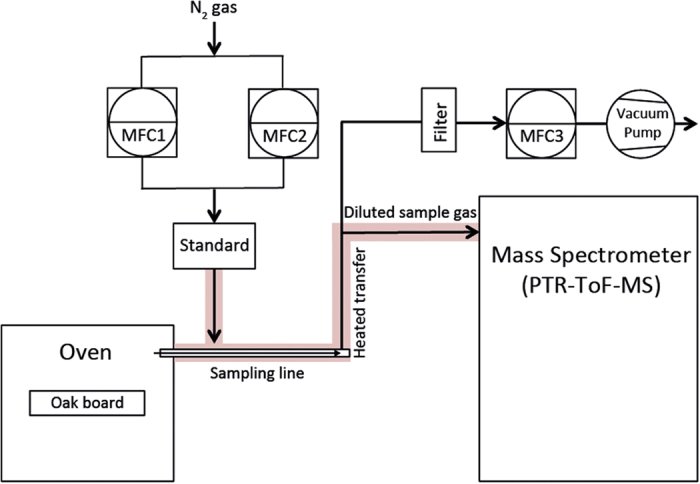
Experimental setup for sampling of oak volatiles generated during the toasting process. Volatiles were continuously drawn into the sampling line by the vacuum pump flow and diluted according to the toasting temperature by a nitrogen gas flow containing the mass calibration standard. The vacuum pump and dilution gas flows were controlled by mass flow controllers (MFC1-3). MFC capacities were MFC1: 5 L, MFC2: 0.2 L and MFC3: 10 L. MFC2 was included in the dilution gas supply to permit accurate adjustment and control of flow via a low capacity MFC.

Volatiles released from the oak board during toasting were measured as shown in Fig. 7. This sampling configuration was adapted from Wellinger^35^ using a sampling line with an integrated dilution port. The sampling line was inserted into the oven to actively sample and deliver toasting process-gas to the inlet of the mass spectrometer. The process-gas was diluted with nitrogen gas (in the sampling line and outside the oven) at a specific ratio for each toasting temperature to avoid condensation of sampled volatiles in the transfer tube, maintain stable and reproducible ionization conditions in the proton transfer reaction drift tube and to minimize detector saturation. The dilution factors were decreased at lower temperatures to retain sensitivity. Appropriate dilution factors were determined experimentally by monitoring the stability of the primary ion (H_3_O^+^) during preliminary toasting trials. The gas fractions sampled at each toasting treatment were 92.8% (175 ^o^C), 27.2% (200 ^o^C) and 6.8% (225 ^o^C), resulting in dilution factors of 1.1, 3.7 and 14.6 respectively. The calibrated sampling flow into the mass spectrometer inlet was 249 standard cubic centimetres per minute (sccm) for all toasting experiments. The gas flows were controlled by mass flow controllers (Bronkhorst, Ruurlo, The Netherlands) and quantified for each dilution setting with a calibrated bubble flowmeter (Sigma-Aldrich (St. Louis, USA) and corrected according to operating temperature and atmospheric pressure. The vacuum pump flow was controlled by a 10 L mass flow controller (MFC3 in Fig. 7). The dilution gas was controlled by two mass flow controllers of different capacity (MFC1: 5 L and MFC2: 0.2 L). MFC2 enabled fine control of the dilution gas supply. Transfer lines (0.25 in. Silcosteel-CR, 316 grade by Restek) were heated to 120 ^o^C and insulated to minimize condensation and memory-effects. A standard (2-isobutyl-3-methylpyrazine, molecular weight: 150.22 u, Sigma-Aldrich, St Louis, USA) was added to the dilution gas stream for high mass range calibration of the mass spectrometer.

### Proton transfer reaction time-of-flight mass spectrometry

A PTR-ToF-MS 8000, mass resolution > 5,000 (FWHM) from Ionicon Analytik GmbH (Innsbruck, Austria) was used. The drift tube voltage was set at 600 V, drift tube pressure at 2.3 mbar (E/N 138 Td) and drift tube temperature at 90 °C. A 5  min blank was recorded before and after each toasting experiment to allow for background correction and to check for memory-effects. The time-of-flight extraction frequency was 50 kHz with a data acquisition rate corresponding to one mass-spectrum recorded every 2 s.

### Mass spectral data processing

PTR-TOF Data Analyzer (v 4.21)^36^ was used for data analysis. The intrinsic H_3_^18^O^+^ and H_3_^18^O^+^(H_2_O) signals (21.022 m/z and 39.033 m/z) as well as the standard 2-isobutyl-3-methylpyrazine (protonated form): [C_9_H_15_N_2_]^+^, 151.123 m/z) were used for mass axis calibration and reference peak shape. Output files were processed according to a targeted mass list based on the protonated form of known oak aroma compounds^9^,^37^,^38^,^39^,^40^,^41^. Spectra were averaged during data processing creating result files with a ten second resolution to increase both accuracy and precision of peak fitting. Ion count rates were normalized to 10^6^ H_3_O^+^ primary ions within the software. Ion counts were then background corrected and adjusted for the dilution factor utilized at each toasting temperature.

### Compound and m/z Selection

A targeted approach was used selecting seven ion traces (m/z values) tentatively identified as the [M+H]^+^ peaks for key aroma compounds derived from the three main aroma precursors in oak wood i.e. polysaccharides (hemicellulose and cellulose), lignin and lipids. These diverse compounds (furanic aldehydes, volatile phenols, phenolic aldehydes, oak lactones and enolic compounds), are widely referred to in the literature and largely dictate the organoleptic quality of toasted oak and sensory impact on oak-aged wine^8^,^42^. Isomeric species cannot be differentiated by PTR-ToF-MS thus, the *cis* and *trans* isomers of oak lactone are collectively analyzed as the sum of both isomeric forms. Furthermore, as some lactones have been reported to fragment extensively in proton-transfer-reaction mass spectrometry^43^, oak lactone fragmentation was checked under the analytical conditions used (Supplementary Table 11). Although fragmentation was observed sufficient analytical sensitivity was still achieved. Maltol and 5-hydroxymethylfurfural have the same chemical formulae thus results reported (at this m/z) relate to the contribution of both compounds. We assign the signal at 127 m/z to the sum of both 5-hydroxymethylfurfural and maltol. Furfural was generated in large concentrations during the toasting process, resulting in detector saturation for this m/z. Consequently, furfural intensities reported were calculated from the isotopic ratio for the ^13^C isotope.

### Data Analysis and Statistics

The time-intensity profile for each (m/z) studied (intensity shown as background and dilution corrected normalized counts per second) was plotted to elucidate the compound evolution profile. As the proton-transfer-reaction mass spectral signal is proportional to compound concentration^44^, the area under the curve for each ion trace was calculated (numerical integration in discrete time-intervals of 10 s corresponding to the data processed by PTR-ToF data analyzer). The integrated area was used to provide insight on the differences between oak source, individual boards and temperatures with regards to the total volatilized amount for each compound. The two oak sources and three toasting temperatures created six datasets, each containing four samples. All 24 samples were tested for significant differences using ANOVA followed by Tukey’s test (p < 0.05). Principal component analysis and agglomerative hierarchical clustering based on dissimilarities (Euclidean distances) and Ward’s agglomeration method was performed on the peak areas for the selected compounds. These chemometric approaches were used to visualize correlations and between-board variation and explore classification by statistical means. Statistical analysis was performed using XLstat (Addinsoft, Paris, France, version 2015.2.01.16529).

## Additional Information

**How to cite this article**: Farrell, R. R. *et al.* Real-Time Mass Spectrometry Monitoring of Oak Wood Toasting: Elucidating Aroma Development Relevant to Oak-aged Wine Quality. *Sci. Rep.* **5**, 17334; doi: 10.1038/srep17334 (2015).

## Supplementary Material

Supplementary Information

## Figures and Tables

**Table 1:** List of mass peaks, chemical formulae, tentative identification of compounds and mean peak areas by oak source and toasting temperature.

measured mass	exact mass	error	mass accuracy	tentative identifiation	chemical formula	two-way ANOVA		peak areas^a^						
(Da)	(Da)	(mDa)	(ppm)			S^b^	T^b^	SxT^b^	FO-175	FO-200	FO-225	AO-175	AO-200	AO-225
97.031	97.0284	2.8	28	furfural	C_5_H_4_O_2_^+^	n.s.	***	n.s	3.7E + 02 ± 2.5E + 02^b^	2.5E + 03 ± 8.9E + 02^b^	2.0E + 04 ± 3.9E + 03^a^	4.3E + 02 ± 2.0E + 02^b^	3.2E + 03 ± 1.7E + 03^b^	2.5E + 04 ± 1.7E + 04^a^
111.048	111.0441	3.6	33	5-methylfurfural	C_6_H_6_O_2_^+^	n.s.	***	n.s.	2.5E + 01 ± 2.7E + 01^b^	2.8E + 02 ± 2.4E + 02^b^	2.9E + 03 ± 2.6E + 03^a^	2.1E + 01 ± 1.3E + 01^b^	2.5E + 02 ± 2.2E + 02^b^	2.7E + 03 ± 2.6E + 03^a^
125.064	125.0597	4.6	36	guaiacol	C_7_H_8_O_2_^+^	*	***	**	6.1E-01 ± 5.2E-01^c^	6.5E + 00 ± 3.6E + 00^c^	6.8E + 01 ± 2.8E + 01^b^	1.3E + 00 ± 5.4E-01^c^	8.6E + 00 ± 5.1E + 00^c^	9.9E + 01 ± 4.6E + 01^a^
127.041	127.0390	2.3	18	5-hydroxymethylfurfural/maltol	C_6_H_6_O_3_^+^	***	***	***	6.4E + 00 ± 5.5E + 01^d^	5.5E + 01 ± 1.5E + 01^cd^	4.1E + 02 ± 1.2E + 02^b^	8.4E + 00 ± 4.7E + 00^d^	8.2E + 01 ± 3.3E + 01^c^	6.3E + 02 ± 8.9E + 01^a^
153.056	153.0546	1.2	8	vanillin	C_8_H_8_O_3_^+^	*	***	*	2.7E + 00 ± 2.3E + 00^c^	3.3E + 01 ± 2.7E + 01^c^	3.5E + 02 ± 1.6E + 02^b^	4.8E + 00 ± 1.9E + 00^c^	5.7E + 01 ± 4.7E + 01^c^	6.9E + 02 ± 5.4E + 02^a^
157.122	157.1223	0.2	1	oak lactone	C_9_H_16_O_2_^+^	***	***	***	6.7E + 00 ± 4.5E + 00^c^	1.9E + 01 ± 8.6E + 00^b^^c^	7.9E + 01 ± 3.2E + 01^b^	1.8E + 01 ± 1.2E + 01^b^^c^	5.1E + 01 ± 2.0E + 01^b^^c^	2.2E + 02 ± 1.3E + 02^a^
165.092	165.0910	1.1	7	eugenol	C_10_H_12_O_2_^+^	***	***	***	4.7E-01 ± 4.6E-01^c^	3.2E + 00 ± 2.2E + 00^c^	5.1E + 01 ± 1.8E + 01^b^	1.1E + 00 ± 7.3E-01^c^	9.7E + 00 ± 6.5E + 00^c^	1.2E + 02 ± 8.3E + 01^a^

^a^peak areas were calculated by numerical integration at discrete ten second time intervals, FO = French oak, AO = American oak.

^b^Oak source effect (S), temperature effect (T) and source x temperature interaction (SxT) significant at the *P < 0.05, **0.01, ***0.001 level, n.s. not significant. Peak areas given are averages for the four boards within each category. Data followed by different letters (in a row) are significantly different according to ANOVA at the P < 0.05 level.
