# Supplementary material for: Real-Time Mass Spectrometry Monitoring of Oak Wood Toasting: Elucidating Aroma Development Relevant to Oak-aged Wine Quality
Source: Sci Rep. 2015 Nov 27;5:17334. doi: 10.1038/srep17334 (PMC4661485; doi:10.1038/srep17334)
Supplement: Supplementary Information [file srep17334-s1.pdf]

Real-Time Mass Spectrometry Monitoring of Oak Wood Toasting: Elucidating Aroma Development Relevant to Oak-aged Wine Quality

SUPPLEMENTARY INFORMATION

Authors:

Ross R. Farrell[1], Marco Wellinger[2], Alexia N. Gloess[2], David S. Nichols[3], Michael C. Breadmore[1], Robert A. Shellie[1] and Chahan Yeretzian[2]

Affiliations:

1. Australian Centre for Research on Separation Science (ACROSS), University of Tasmania, Hobart, Tasmania 7001, Australia

2. Zurich University of Applied Sciences, Institute of Chemistry and Biological Chemistry, 8820 Wädenswil, Switzerland

3. Central Science Laboratory, University of Tasmania, Private Bag 74, Hobart, Tasmania 7001, Australia

Correspondence to: Ross Farrell, Australian Centre for Research On Separation Science, University of Tasmania, Private Bag 75, Hobart, 7001, Australia. ross.farrell@utas.edu.au

Supplementary Information

1) Supplementary Tables

Supplementary Table 1: Moisture content and density properties for the oak staves measured prior to the toasting process.

French Oak			American Oak		
Board	Moisture content (%)	Oven-dry density (g/cm^3)	Board	Moisture content (%)	Oven-dry density (g/cm^3)
1	10.8	0.689	1	9.5	0.707
2	11.1	0.755	2	8.4	0.736
3	10.0	0.682	3	9.6	0.771
4	9.8	0.701	4	10.7	0.782
Average	10.4	0.707	Average	9.5	0.749
Standard deviation	0.6	0.033	Standard deviation	0.9	0.034

Supplementary Table 2: Summary of temperature effects according to ANOVA (least square means) for each compound.

	Furfural	5MF	HMF	Vanillin	Eugenol	Guaiacol	Lactone
225	22413.690 a	2774.267 a	521.328 a	518.353 a	74.999 a	95.993 a	151.628 a
200	2854.985 b	266.119 b	68.469 b	45.297 b	5.867 b	8.125 b	34.919 b
175	399.158 b	22.803 b	7.420 c	3.730 b	0.878 b	0.877 b	12.296 b
Pr > F	<0.0001	<0.0001	<0.0001	<0.0001	<0.0001	<0.0001	<0.0001
Significant	Yes	Yes	Yes	Yes	Yes	Yes	Yes

Data followed by different letters (in a column) are significantly different according to ANOVA at the $P < 0.05$ level.

Supplementary Table 3: Summary of pairwise comparisons for species for furfural according to Tukey's Honestly Significantly Different test.

Category	LS means(Furfural)	Groups
AO	9574.848	A
FO	7537.041	A

Categories (rows) with different letters are significantly different according at the $P < 0.05$ level.

Supplementary Table 4: Summary of pairwise comparisons for species for 5-methylfurfural (5MF) according to Tukey's Honestly Significantly Different test.

Category	LS means(5MF)	Groups
FO	1064.329	A
AO	977.797	A

Categories (rows) with different letters are significantly different according at the $P < 0.05$ level.

Supplementary Table 5: Summary of pairwise comparisons for species for 5-hydroxymethylfurfural (HMF) according to Tukey's Honestly Significantly Different test.

Category	LS means(HMF)	Groups	
AO	240.532	A	
FO	157.613		B

Categories (rows) with different letters are significantly different according at the P < 0.05 level.

Supplementary Table 6: Summary of pairwise comparisons for species for vanillin according to Tukey's Honestly Significantly Different test.

Category	LS means(Vanillin)	Groups	
AO	249.696	A	
FO	128.557		B

Categories (rows) with different letters are significantly different according at the P < 0.05 level.

Supplementary Table 7: Summary of pairwise comparisons for species for eugenol according to Tukey's Honestly Significantly Different test.

Category	LS means(Eugenol)	Groups	
AO	36.205	A	
FO	18.291		B

Categories (rows) with different letters are significantly different according at the P < 0.05 level.

Supplementary Table 8: Summary of pairwise comparisons for species for guaiacol according to Tukey's Honestly Significantly Different test.

Category	LS means(Guaiacol)	Groups	
AO	45.117	A	
FO	24.880		B

Categories (rows) with different letters are significantly different according at the P < 0.05 level.

Supplementary Table 9: Summary of pairwise comparisons for species for oak lactone according to Tukey's Honestly Significantly Different test.

Category	LS means(Lactone)	Groups	
AO	97.634	A	
FO	34.929		B

Categories (rows) with different letters are significantly different according at the P < 0.05 level.

Supplementary Table 10: Summary of pairwise comparisons for each species x temperature category according to Tukey's Honestly Significantly Different test.

Category	LS means(Lactone)	Groups		
AO*225	223.918	A		
FO*225	79.338		B	
AO*200	51.076		B	C
FO*200	18.763		B	C
AO*175	17.906		B	C
FO*175	6.686			C

Categories (rows) with different letters are significantly different according at the P < 0.05 level.

Supplementary Table 11: Fragmentation of oak lactone under proton-transfer-reaction mass spectrometry conditions utilized in this work.

Compound	Molecular weight	Relative abundance[a] of major ions[b] [(relative abundance)]			
Oak lactone	156	**157** (100)	139 (48)	97 (11)	55 (10)

[a] data presented as the background corrected counts per second normalized to the most abundant mass fragment (relative abundance 100). All other intensities are calculated relative to the most abundant mass fragment.
[b] Mass fragments with intensity less than 10 not shown. $[M+H]^+$ in bold.
